# Genome-Wide Discovery and Characterization of the Auxin Response Factor (ARF) Gene Family in *Avicennia marina* That Regulates Phytohormone Levels and Responds to Salt and Auxin Treatments

**DOI:** 10.3390/biology14121774

**Published:** 2025-12-11

**Authors:** Quaid Hussain, Muhammad Azhar Hussain, Yingying Li, Qi Zhang, Chenjing Shang, Mostafa A. Abdel-Maksoud, Salman Alrokayan, Abdulaziz Alamri

**Affiliations:** 1 Guangdong Provincial Key Laboratory for Plant Epigenetics, Shenzhen Key Laboratory of High-Efficiency Utilization of Light in Plants, College of Life Science and Oceanography, Shenzhen University, Shenzhen 518060, China; quaid_hussain@yahoo.com (Q.H.); 2410173052@mails.szu.edu.cn (Q.Z.); 2 College of Physics and Optoelectronic Engineering, Shenzhen University, Shenzhen 518060, China; 3 Research Chair of Biomedical Applications of Nanomaterials, Biochemistry Department, College of Science, King Saud University, P.O. Box 2455, Riyadh 11451, Saudi Arabia; 4 Biochemistry Department, College of Science, King Saud University, P.O. Box 2455, Riyadh 11451, Saudi Arabia

**Keywords:** phytohormone contents, phylogenetic analysis, gene ontology, microRNAs, qRT-PCR

## Abstract

Auxin response factors (ARFs) are crucial components of auxin signaling, playing a vital role in plant growth, development, hormone regulation, and stress responses. Salinity influences plant growth and development; however, *Avicennia marina* exhibits remarkable salt tolerance. This study analyzed *Avicennia marina ARF* genes (*AmARFs*) and their roles in responding to salt and indole-3-acetic acid (IAA) stress. The results indicated that across 5–15 days, endogenous IAA and abscisic acid (ABA) levels in *A. marina* leaves showed significant, time-dependent changes under salt and IAA treatments, with IAA fluctuating around 2.0–3.3 µg g^−1^ FW and ABA rising sharply under combined high-salt + IAA conditions (AS25), reaching up to ~25 µg g^−1^ FW (*p* < 0.05). This is the first genome-wide identification of 41 *ARF* genes in *Avicennia marina* with expression responses to combined salt and auxin treatments. We identified 41 *AmARF* genes spread across 23 chromosomes. These genes are divided into four groups according to their phylogenetic relationships. Their coding regions encode amino acids from 361 to 1264, and the number of exons varies from 2 to an unspecified upper limit of 25. Examining these gene promoters revealed various hormone- and stress-response elements, with each gene containing distinct response elements. Sixteen miRNAs can inhibit various *ARF* genes, while protein–protein interactions and 3D structures offered valuable insights into AmARF proteins. GO enrichment analysis revealed that all 41 *AmARFs* are involved in the auxin-activated signaling pathway and are also involved in cell division. According to the expression experiments, 11 randomly selected genes showed predominantly upregulation in response to salt and IAA stressors compared with controls. These findings extend our understanding of the functional roles of *AmARFs* in stress responses. The systematic annotation of AmARF family genes offers candidate genes for future functional validation, which may help elucidate the precise roles of *AmARFs* in stress responses.

## Simple Summary

Mangrove plants like *Avicennia marina* (*A. marina*) can live in very salty environments. We studied a group of genes called ARFs, which help control plant hormones and stress responses. In this work, we identified all 41 ARF genes in *A. marina* and examined their features. We also measured two important hormones, IAA and ABA, under salt and IAA treatments, and found clear changes, especially a strong rise in ABA under high salt. Several ARF genes showed increased activity during these treatments. Our results provide the first complete overview of *ARF* genes in this species and help explain how *A. marina* responds to salt stress.

## 1. Introduction

Mangroves are woody plants that grow in the intertidal zone along tropical and subtropical coastlines and estuaries. They are vital for shielding coastal regions from storm surges, reinforcing the shoreline, and maintaining the ecological balance of these sensitive habitats [1]. *Avicennia marina* is a key pioneering species within the mangrove ecosystem, renowned for its exceptional resilience to high salinity. This makes it an important subject for investigating plant salt tolerance mechanisms [1,2]. Most earlier studies primarily emphasize physiology, biochemistry, or the functional roles of coding genes [3]. Therefore, investigating the molecular mechanisms that enable *A. marina* to tolerate salt is essential for understanding how mangroves thrive in coastal intertidal zone habitats.

Indole-3-acetic acid (IAA), the primary auxin, is crucial for plant growth and development, as it responds to cellular signals and environmental cues [4,5]. These functions primarily involve gene regulation involving auxin response factor (ARF) proteins. ARFs decode the IAA signal to regulate gene expression, aided by auxin/indole-3-acetic acid (Aux/IAA) proteins [6,7]. Auxin controls various facets of plant growth and stress response via a well-understood signaling pathway that includes Aux/IAA repressors, the SCFTIR1/AFB complex, and subsequent ARFs [8,9]. ARF proteins generally feature a DNA-binding domain, a central region that influences transcriptional activation or repression, and a conserved PB1 domain responsible for mediating protein–protein interactions [10]. While the fundamental auxin signaling pathway is mostly conserved across plant species, ARF gene families frequently undergo lineage-specific expansion and functional divergence [11]. Genetic research has revealed two primary groups of transcription factors that play a role in auxin signaling: ARFs and Aux/IAA proteins [12,13]. ARFs are plant-specific proteins, typically composed of an N-terminal DNA-binding domain (DBD), a non-conserved middle region (MR), and a C-terminal domain (CTD) involved in dimerization and protein interactions. They regulate auxin-responsive gene expression by binding to specific promoter regions, such as the auxin response element (TGTCTC). Moreover, ARFs can form dimers through interactions with Aux/IAA proteins [14,15]. Many ARF family members have been identified and studied across various plant species since the first ARF gene was cloned in *Arabidopsis thaliana*, or *AtARF1*. Genomic analyses have revealed the number of members in different species: 81 in Alfalfa (*Medicago sativa*) [16], 41 in kiwifruit (*Actinidia chinensis*) [17], 35 in *Populus trichocarpa* [7], 29 in Mango (*Mangifera indica* L.) [18], 19 in sweet orange (*Citrus sinensis*) [19], and 17 in Peach (*Prunus persica* L.) [20]. The variation in *ARF* gene numbers among species may reflect differences in genome size, duplication history, and ecological adaptation. In mangrove species such as *A. marina*, previous genomic studies have reported lineage-specific expansion of stress-responsive genes driven by segmental duplications and selective pressures associated with high salinity and tidal environments [1]. Such evolutionary processes may result in an *ARF* gene repertoire that differs from those of terrestrial plants, potentially supporting the unique physiological and hormonal regulation required for survival in saline coastal habitats. Previous research has shown that *ARFs*, as transcriptional regulators, directly impact auxin signal transduction [21] and engage in different stress responses and hormonal interactions in Arabidopsis [22], rice [23], and citrus [19]. In Arabidopsis, several *ARF* genes are involved in responses to abiotic stress [22]. In tomatoes, the *SlARF* gene affects responses to salt, drought, and flooding stresses. At the same time, *SlARF7* is involved in the interaction between auxin and gibberellic acid (GA) signaling during fruit and plant development [24]. Gibberellic acid (GA), an essential plant hormone that regulates growth, interacts with auxin and other hormones to coordinate developmental responses during stress conditions [25]. Besides auxin, abscisic acid (ABA) plays a key role as a stress hormone that influences stomatal function, osmotic regulation, and signaling during salinity stress. Since salinity can shift the balance between auxin and ABA, measuring ABA levels is crucial for understanding how these hormones interact in *A. marina* when exposed to salt and external IAA treatments [26]. Similarly, several *ARF* genes play a role in how plants respond to abiotic stresses, such as drought, salt, and cold [23]. In chickpea (*CaARFs*) [27] and kiwifruit (*AcARFs*) [13], genes are essential regulators of abiotic stress responses.

Recently, microRNAs (miRNAs) have been identified as active players in environmental stress responses by modulating the expression of genes involved in auxin response [28,29]. In Arabidopsis, miR167 targets *AtARF6/8* and miR160 targets *AtARF10/16/17* [30,31]. miR160 and miR167 support normal development during stress by regulating the expression of their ARF gene targets [32]. Although *A. marina* is a halophytic tree with remarkable salt tolerance, its ARF gene family has not been thoroughly studied. There has been no comprehensive research identifying *ARF* genes in their genomes, examining their structural and evolutionary features, or exploring their roles in phytohormone regulation during salt and auxin treatments. This knowledge gap limits our understanding of how auxin-mediated transcription influences the species’ stress-adaptation mechanisms. Consequently, conducting a genome-wide analysis of *ARF* genes in *A. marina* is essential to uncover their functions in growth control and saline resilience. These findings suggest that ARF genes play a crucial role in regulating plant growth, development, and responses to abiotic stress. *Avicennia marina* is a mangrove species that thrives in hypersaline environments, thereby helping preserve coastal biodiversity and ecosystems. However, the mechanisms and regulators underlying its response to salinity and IAA, both individually and in combination, are poorly understood.

The ARF gene family has not yet been identified in *A. marina*. This study represents the first comprehensive genome-wide effort to identify *ARF* genes in *A. marina’s* genome. The main goal was to locate and characterize the 41 *ARF* genes, as well as analyze phytohormone content and expression levels under four treatments: 15‰ salt (S15, the control, since *A. marina* tolerates salt), 25‰ salt (S25), 50 µM IAA with 15‰ salt (A15), and 50 µM IAA with 25‰ salt (AS25). Although *Avicennia marina* is naturally salt-tolerant, salinity has a substantial impact on auxin biosynthesis, transport, and signaling. Auxin interacts with ABA under salt stress to regulate osmotic adjustment, ion homeostasis, and root growth, including lateral root formation. Therefore, applying both salt and exogenous IAA allows identification of *ARF* genes responsive to salt, auxin, or their combined effects. Bioinformatics methods were employed to investigate multiple aspects of *ARF* gene evolution in *A. marina*, aiming to gain a deeper understanding. These aspects included analyzing physicochemical properties, subcellular locations, phylogenetic relationships, conserved motifs, gene structures, cis-regulatory elements, miRNA interactions, gene ontology annotations, duplication events, chromosomal organization, and expression patterns of ARF homologs. This study focuses on characterizing and analyzing the expression of the ARF family and phytohormone contents in *A. marina* leaves. The goal is to establish a theoretical basis for future research on how the ARF family responds to salt and IAA treatments in *A. marina* plants. Therefore, this study aimed to identify and characterize the ARF gene family in *Avicennia marina*, investigate its promoter elements and potential miRNA regulation, and evaluate the expression patterns of these genes under salt and auxin treatments.

## 2. Materials and Methods

### 2.1. Plant Materials and Stress Conditions

The study utilized 9-month-old *Avicennia marina* seedlings sourced from Yuehui Planting and Breeding on Xunzhou Island, China (117°01.7′ E, 23°34.4′ N). The irrigation experiment lasted 15 days, beginning on April 29, 2025. Four treatments were applied via soil irrigation: 15‰ salt (S15, control), 25‰ salt (S25), 50 µM IAA + 15‰ salt (A15), and 50 µM IAA + 25‰ salt (AS25). Individual plants served as experimental units, with 10–12 plants per replicate and three biological replicates per treatment. IAA and ABA treatments were applied at the start of the experiment, and leaf samples were collected 5, 10, and 15 days after treatment for gene expression and phytohormone analyses.

### 2.2. Phytohormone (IAA and ABA) Content Determination

We measured the levels of two phytohormones, IAA and ABA, at 5, 10, and 15 days after applying the treatments: 15‰ salt (S15, used as the control since *A. marina* is salt-tolerant), 25‰ salt (S25), 50 µM IAA + 15‰ salt (A15), and 50 µM IAA + 25‰ salt (AS25). The endogenous IAA and ABA hormone contents were extracted and purified from samples stored at −80 °C. Approximately 0.4 g of the sample was initially ground in liquid nitrogen. Extraction was conducted using 1.5 mL of pre-cooled 80% methanol aqueous solution. After crushing, the samples were extracted overnight at 4 ◦ C. Next, the samples were centrifuged at 8000× *g* for 10 min, and the resulting supernatant was collected. The remaining residues were extracted with 0.5 mL of an 80% aqueous methanol solution for two hours. After centrifugation, the supernatant was collected again, and the two portions were combined. The remaining organic phase (methanol aqueous solution) was evaporated to dryness under nitrogen (N_2_) at 40 °C until all the organic phase had been removed. Subsequently, 2 mL of petroleum ether was added to the samples for further extraction and decolorization, repeating this step three times. A 1 mol L^−1^ aqueous citric acid solution was added to the lower aqueous phase to adjust the pH to 2–3. After adding 2 mL of ethyl acetate for extraction on two occasions, the upper organic phase was transferred to a new EP tube and dried under nitrogen (N_2_).

Additionally, 0.2 mL of mobile phase was added to facilitate proper dissolution and mixing. The samples were then filtered using a syringe filter before analysis of IAA and ABA. Separation and quantification of IAA and ABA were performed using a high-performance liquid chromatography (HPLC) system (Waters 2695, Milford, MA, USA). This system utilized a C18 reversed-phase column (250 mm × 4.6 mm, 5 μm) for effective separation. This process was conducted at 35 °C with a flow rate of 1 mL/min and an injection volume of 10 μL. For ABA, the mobile phase was a 1% acetic acid solution in water mixed with methanol (60:40 *v*/*v*), with detection monitored at 254 nm. In comparison, the mobile phase for IAA consisted of 0.1% phosphoric acid in water, combined with acetonitrile (85:15 *v*/*v*), and detection was monitored at 210 nm [33].

### 2.3. Identification and Physicochemical Aspects of ARF Members in A. marina

The most recent genome data for *Avicennia marina* were retrieved from DRYAD (https://datadryad.org/dataset/doi:10.5061/dryad.3j9kd51f5, accessed on 4 June 2025) to identify its *ARF* gene members [34]. The hidden Markov model (HMM) files for the ARF domain (PF06507) were retrieved from the Pfam database (http://pfam-legacy.xfam.org/, accessed on 4 June 2025). Then, the HMMER program was used to identify the ARF proteins in the *A. marina* genome. Finally, the Auxin_resp (PF06507) domain of all putative ARF proteins was determined through the conserved domain database Batch CD-Search (https://www.ncbi.nlm.nih.gov/Structure/bwrpsb/bwrpsb.cgi, accessed on 4 June 2025). We employed TBtools-II (Toolbox for Biologists) version 2.225 to perform a BLASTp (Basic Local Alignment Search Tool for Protein) search to identify homologous ARF protein sequences in *A. marina*, using the default settings. This analysis utilized PF06507, the relevant homologous file, and the complete genome protein sequences (Table S4). The physicochemical properties of the AmARF protein sequences were evaluated using TBtools-II (Toolbox for Biologists) v2.225. Using the Cell-PLoc 2.0 tool (Protein Subcellular Localization Prediction http://www.csbio.sjtu.edu.cn/bioinf/Cell-PLoc-2/, accessed on 4 June 2025), we identified the subcellular localization of 41 genes in *A. marina*. The Phyre2 online platform predicted secondary and three-dimensional structures (https://www.sbg.bio.ic.ac.uk/phyre2/html/page.cgi?id=index, accessed on 4 June 2025). To predict transmembrane domains in AmARF proteins, the TMHMM 2.0 tool was used (https://services.healthtech.dtu.dk/services/TMHMM-2.0/, accessed on 4 June 2025).

### 2.4. Phylogenetic Analysis, Conserved Motifs, and Gene Structure Assessment

The protein sequences of ARF genes from *Avicennia marina* (jg), *Kandelia obovata* (GWH), and *Arabidopsis thaliana* (AT) were used in the phylogenetic analysis (Table S5). Multiple sequence alignment of ARF protein sequences was performed in MEGA12 (www.megasoftware.net, accessed on 5 June 2025) using the MUSCLE alignment algorithm. The phylogenetic tree was generated using the Neighbor-Joining (NJ) method with the Poisson model and 1000 bootstrap replicates. The final tree was visualized and refined using iTOL (https://itol.embl.de/, accessed on 5 June 2025).

The amino acid sequences for all 41 *ARF* genes were examined using MEME Suite v5.5.4 (https://meme-suite.org/meme/, accessed on 5 June 2025) to detect conserved motifs. The genome annotation information from the original GFF file was analyzed to identify the gene structure of *ARFs.* A conserved-domain analysis was performed by querying the Pfam database in NCBI-CDD (Conserved Domain Database), resulting in a corresponding hit-data file. The full-length protein sequence alignment was performed using the MUSCLE algorithm in MEGA-12, and a Newick file was generated as described above. The complete analysis used TBtools v2.225.

### 2.5. Putative Promoter Region Analysis

The screened *A. marina* ARF family genes were analyzed using TBtools v2.225 to extract 2500 bp upstream promoter regions, a commonly used length that captures most proximal regulatory elements; however, we acknowledge that more distal regulatory elements beyond this region may not be included. These extracted promoter sequences were submitted to the PlantCARE database (https://bioinformatics.psb.ugent.be/webtools/plantcare/html/, accessed on 5 June 2025) for further analysis. The TBtools v2.225 software was used to visualize and predict various regulatory elements involved in hormone, growth, development, and stress responses. The cis-acting elements in this family were classified based on their functional characteristics, utilizing relevant literature.

### 2.6. Proposed miRNA Targeting ARFs and Evaluation of Their Functions

The psRNATarget website was utilized to identify miRNA target sites. We submitted the CDSs of all *ARF* genes to the website (https://www.zhaolab.org/psRNATarget/, accessed on 6 June 2025), employing *Populus trichocarpa* as a reference and using the default parameter settings. A diagram illustrating the interaction between miRNA, target genes, and *AmARF* genes was created with https://www.bioinformatics.com.cn/plot_basic_miRNA_target_network_plot_197, accessed on 6 June 2025.

### 2.7. Gene Ontology and Enrichment Analysis

To determine the GO enrichment, the Gene Ontology (GO) data for *AmARF* genes were sourced from DRYAD (https://datadryad.org/dataset/doi:10.5061/dryad.3j9kd51f5, accessed on 6 June 2025) [34]. Enrichment analyses were performed by retrieving the GO-basic ontology data from the REVIGO (Reduce + visualize Gene Ontology) online platform (http://revigo.irb.hr/).

### 2.8. Collinearity, Ka/Ks Ratios, and Protein Similarities of AmARFs

The collinearity analysis of *ARF* genes in *Avicennia marina* was conducted in conjunction with two other plant species, *Kandelia obovata* and *Arabidopsis thaliana*, using the Circletto online software (https://bat.infspire.org/circoletto/, accessed on 6 June 2025). The AmARF protein sequences were typically used as query sequences, while protein sequences from the other two species served as databases. In the blast analysis, different colors—blue, green, orange, and red—represented protein sequence identities of <25%, <50%, <75%, and >75%, respectively. Finally, Circos was computed and visualized based on the blast scores. The substitution rates of Ka and Ks, along with the Ka/Ks ratios and protein similarities of *AmARF* at the nucleotide level, were annotated and calculated (Simple Ka/Ks Calculator) using TBtools v2.225 software.

### 2.9. Prediction of Protein–Protein Interaction, 3D Structures, and Chromosomal Localization

The sequences of AmARF proteins were examined using the STRING v12.0 database, configured to generate a complete STRING network allowing up to 10 connections and requiring a minimum interface score of 0.4 (medium confidence). The secondary structures and 3D models of all AmARF proteins were generated using the Phyre2 online server (https://www.sbg.bio.ic.ac.uk/phyre2/html/page.cgi?id=index, accessed on 6 June 2025) with default settings. We utilized the DRYAD (https://datadryad.org/dataset/doi:10.5061/dryad.3j9kd51f5, accessed on 6 June 2025) to identify the genomic locations and protein sequences of 41 *AmARF* genes, and we assessed the distribution of *ARF* genes across chromosomes. Using MapGene2Chromosome (MG2C; http://mg2c.iask.in/mg2c_v2.0, accessed on 6 June 2025, we identified *ARF* genes on the chromosomes of *A. marina*.

### 2.10. Analysis of ARF Genes Using Quantitative Real-Time PCR (qRT-PCR)

According to the product guidelines, the TaKaRa MiniBEST Plant RNA Extraction Kit (Baori Medical Biotechnology Co., Ltd., Beijing, China). It was used to extract total RNA from leaf samples. RNA concentration and purity were assessed with a Thermo Scientific™ NanoDrop™ spectrophotometer (Thermo Fisher Scientific, Waltham, MA, USA). From 1 μg of total RNA, first-strand cDNA was synthesized utilizing the PrimeScript™ RT Reagent Kit (RR047A, Takara) in a Life Technologies PCR machine. A Longgene CFX96 Real-Time System with the TB Green Premix Ex Taq II Kit (CN830B, Takara) was used for quantitative PCR. Each reaction contained 2 μL of diluted cDNA template in a total volume of 20 μL, with three technical replicates per sample. The thermal cycling conditions were in accordance with the manufacturer’s specifications. Primer pairs were designed from the coding sequences of eleven (11) *AmARF* genes using Primer 5 software. Amplification efficiency for each primer pair was determined using a five-point, 10-fold serial dilution of pooled cDNA, yielding efficiencies ranging from 90% to 95%. Melting curve analysis (65–95 °C) confirmed the presence of single, sharp peaks for all reactions, indicating high specificity and the absence of primer dimers. The 18S rRNA gene was used as the internal reference based on its stable expression across salt and auxin treatments in A. marina. Primer sequences are provided in Table S6. Relative gene expression levels were calculated using the 2^(−ΔΔCt)^ method, based on the mean Ct values of technical replicates.

### 2.11. Data Statistics and Analysis

Graphs were generated with GraphPad Prism 9 (https://www.graphpad.com, accessed on 10 June 2025). Data analysis involved one-way ANOVA using OriginPro 9.0, and results are shown as the mean ± standard deviation (SD) of three biological replicates. A least significant difference (LSD) test was conducted at a significance level of *p* < 0.05.

## 3. Results

### 3.1. Changes in IAA and ABA Levels Under Salt and IAA Stress

**Figure 1:**
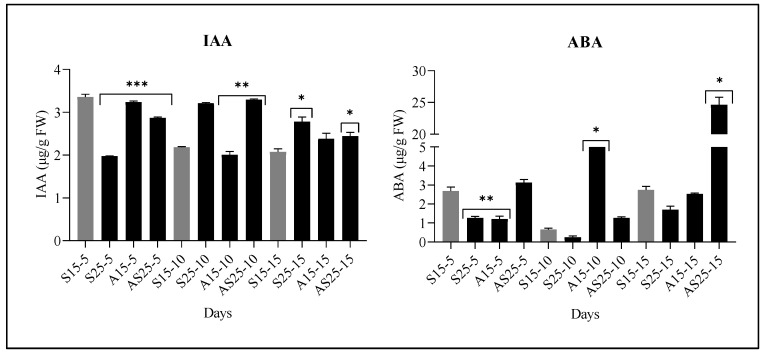
Effects of salt and IAA treatments on IAA and ABA contents in *A. marina* leaves after 5, 10, and 15 days. Treatments included 15‰ salt (S15, control), 25‰ salt (S25), 50 µM IAA with 15‰ salt (A15), and 50 µM IAA with 25‰ salt (AS25). Hormone concentrations are presented as mean ± SD (µg/g FW). Asterisks indicate statistically significant differences compared with the control at each time point (* *p* < 0.05, ** *p* < 0.01, *** *p* < 0.001).

The endogenous IAA and ABA contents in *A. marina* leaves were quantified after 5, 10, and 15 days under four treatments (S15, S25, A15, and AS25). Clear treatment- and time-dependent differences were observed in both hormones (Figure 1). Notable differences (*p* < 0.05) were observed between the control group (S15) and various stress treatments involving salt and IAA, including 25‰ salt (S25), 50 µM IAA with 15‰ salt (A15), and 50 µM IAA with 25‰ salt (AS25). For IAA, the control (S15) maintained levels around ~2.1–3.3 µg/g FW at all three time points. At 5 days, IAA was significantly lower in S25-5 and A15-5 (approximately 2.0–3.2 µg/g FW), while AS25-5 showed a moderate reduction (~2.9 µg/g FW). At 10 days, S25-10 and AS25-10 displayed slightly higher IAA contents (~3.2–3.3 µg/g FW) compared with the control (~2.2 µg/g FW), whereas A15-10 remained lower (~2.0 µg/g FW). By 15 days, all stress treatments showed moderately elevated IAA levels relative to the control: S25-15 (~2.9 µg/g FW), A15-15 (~2.4 µg/g FW), and AS25-15 (~2.4 µg/g FW), with several comparisons showing statistically significant differences. For ABA, the control (S15) showed low baseline levels (approximately 0.7–2.7 µg/g FW). At 5 days, ABA levels decreased under S25-5 and A15-5 (~1.3–1.2 µg/g FW), while AS25-5 showed a slight increase (~3.1 µg/g FW). At 10 days, ABA accumulated strongly in A15-10 (~15.3 µg/g FW), whereas S25-10 remained low (~0.3 µg/g FW). AS25-10 showed a moderate increase (~1.3 µg/g FW). By 15 days, AS25-15 exhibited a marked ABA accumulation (~25 µg/g FW), significantly higher than the control (~2.7 µg/g FW), while S25-15 and A15-15 remained near or slightly below control levels. Overall, hormone levels showed substantial, time-dependent alterations, with exogenous IAA and high salinity (AS25) producing the most pronounced effects, particularly for ABA at later time points (Figure 1).

### 3.2. Identification and Comprehensive Characterization of ARF Members in the Avicennia marina Genome

We discovered ARF genes by searching the Pfam database and acquiring HMM (Hidden Markov Model) profiles for the Auxin-Responsive Factor (ARF) domain (PF06507). During the identification and sequence analysis of the ARF gene family in *A. marina*, we identified 41 *ARF* genes. Subsequently, the physicochemical properties and sequences of the ARF gene family members were analysed (Table 1). AmARF proteins vary in amino acid counts from 361 (jg22944.t1) to 1264 (jg21120.t1), exhibiting molecular weights of 40,412.34 and 139,295.27 daltons (Da). On average, they consist of 807 amino acids and weigh 89,354.98 Da. The coding DNA sequence (CDS) lengths of *AmARFs* range from 1083 bp (jg22944.t1) to 3792 bp (jg21120.t1), resulting in an average CDS length of 2421 bp. The theoretical isoelectric values ranged from 5.3 (jg11799.t1) to 9.39 (jg22944.t1), with an average of 6.73, indicating that different AmARF proteins were weakly acidic and may function in various microenvironments. The instability index of the AmARF family ranged from 40.79 (jg12151.t1) to 66.7 (jg31808.t1); however, the instability index of all genes was greater than 40, indicating that they encode unstable proteins. The aliphatic index of 41AmARF proteins ranged from 64.87 (jg13427.t1) to 82.29 (jg12151.t1), all of which were less than 100, indicating that they were hydrophilic proteins. Subcellular localization predictions aimed to clarify where the family’s expression occurs (Table 1). All predicted *AmARF* proteins were localized to the nucleus, as determined by subcellular localization predictions. As expected for ARF transcription factors, all members were predicted to be nuclear, which aligns with their functional role in binding to auxin response elements (AuxREs) in the promoters of target genes. Additionally, examining the transmembrane domain of the amino acid sequences of AmARFs (Figure S1) reveals a specific protein, jg29466.t1, possesses only a single transmembrane domain. In contrast, the amino acids of these 41 AmARF members are distributed both inside and outside the membrane, indicating that the synthesis of AmARF proteins might directly influence functions without requiring transmembrane transport. Protein sequence alignment was performed (Figure S1), focusing on the Pfam (PF06507) domain and its similarity to the query sequences. The pairwise comparisons of AmARF protein sequences exhibited similarity levels between a low of 20.58% (for jg32038.t1 and jg15264.t1) and a high of 83.88% (for jg3481.t1 and jg14968.t1) (Table S1).

### 3.3. Phylogenetic Relationship of ARF Proteins

**Figure 2:**
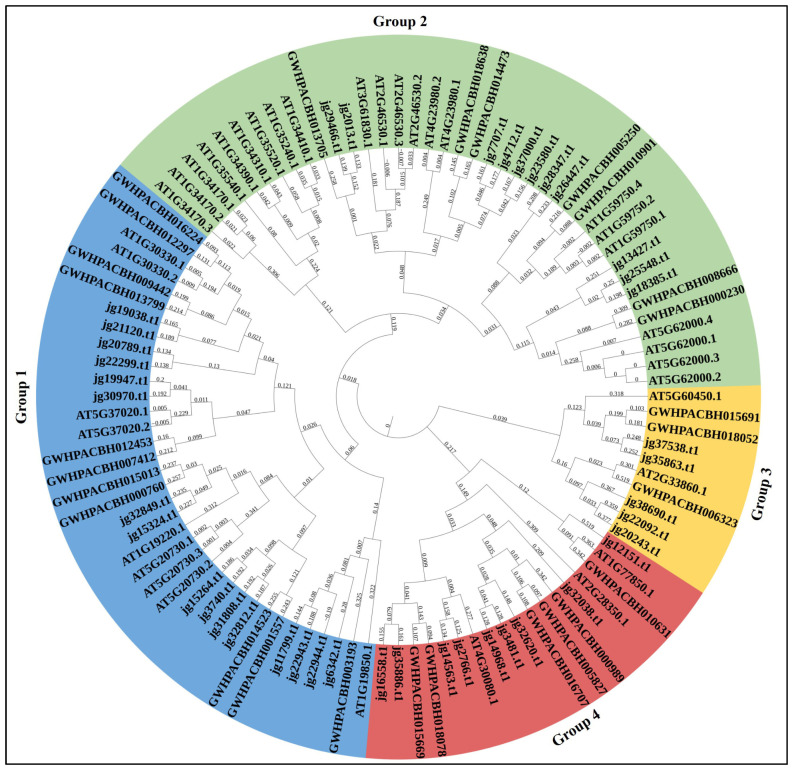
Phylogenetic relationships of ARF proteins from *Avicennia marina* (jg), *Kandelia obovata* (GWH), and *Arabidopsis thaliana* (AT) are depicted. The tree was constructed from 104 full-length ARF amino acid sequences and divided into four main clades (Groups 1–4). These clades are colour-coded: Group 1 (blue), Group 2 (green), Group 3 (yellow), and Group 4 (dark red). Each terminal label contains the species prefix (jg/GWH/AT) and the sequence ID. Branch support values are shown at internal nodes as percentages, indicating the confidence level (using the neighbour-joining method with 1000 bootstrap replicates). The outer rim displays group numbers and the species composition within each group.

To assess the evolutionary relationships among ARF proteins, a phylogenetic tree was constructed from 104 deduced amino acid sequences: 41 from *Avicennia marina* (jg), 27 from *Kandelia obovata* (GWH), and 36 from *Arabidopsis thaliana* (AT). The ARF proteins were resolved into four major clades (Groups 1–4), which are color-coded in the tree: Group 1 (blue; 36 proteins), Group 2 (green; 40 proteins), Group 3 (yellow; 10 proteins), and Group 4 (dark red; 18 proteins) (Figure 2). The composition of each group is as follows. Group 1 comprises 16 *A. marina* (jg), 11 *K. obovata* (GWH), and 9 *A. thaliana* (AT) proteins. Group 2 comprises 11 *A. marina*, 7 *K. obovata*, and 22 *A. thaliana* proteins and is the largest clade. Group 3 contains 5 *A. marina*, 3 *K. obovata*, and 2 *A. thaliana* proteins, while Group 4 contains 9 *A. marina*, 6 *K. obovata*, and 3 *A. thaliana* proteins.

Branch-support values are displayed at internal nodes in Figure 2 to indicate clade robustness. Overall, most major clades are supported by moderate-to-high branch-support values, indicating confidence in the four-group partitioning. To verify the internal consistency of the *A. marina* placements, an *A. marina*-only phylogeny was constructed (jg-only tree). The topology of the jg-only tree is concordant with the combined tree: *A. marina* genes occupy the same four clades and show no major topological conflicts, confirming that the group assignments for the jg genes are stable whether or not homologs from *K. obovata* and *A. thaliana* are included.

### 3.4. Motif Compositions, Conserved Domains, and Gene Structures of AmARF

**Figure 3:**
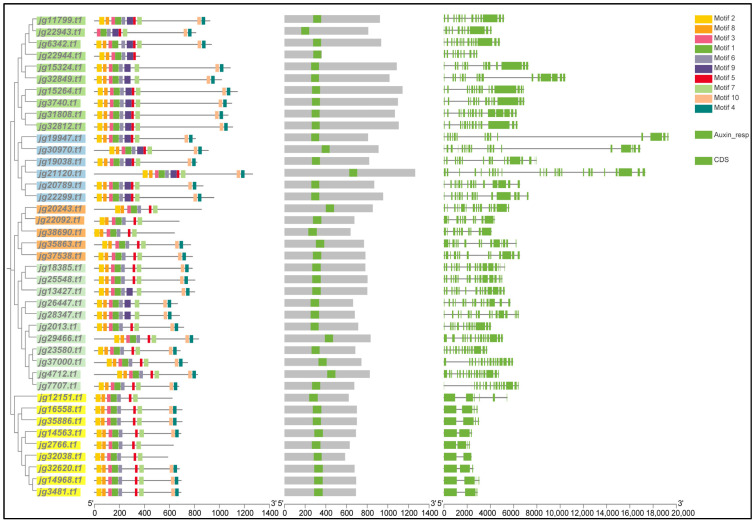
Motif compositions, conserved domains, and gene structures of ARFs in *A. marina*. The left shows colored boxes representing motifs. The green-highlighted Auxin response (Auxin_resp) domain is in the center. Exons (CDS) are represented by green on the right, with introns shown as black lines. The ARF proteins were classified into five groups: Group 1 (green; 10 proteins), Group 2 (blue; six proteins), Group 3 (orange; five proteins), Group 4 (light green; 11 proteins), and Group 5 (yellow; nine proteins).

A phylogenetic tree was made using each sequence of the ARF protein. The ARF proteins were classified into five groups: Group 1 (green; 10 proteins), Group 2 (blue; six proteins), Group 3 (orange; five proteins), Group 4 (light green; 11 proteins), and Group 5 (yellow; nine proteins) (Figure 3). To explore the evolutionary diversity of the AmARF family, we examined the conserved motifs of 41 AmARF proteins using the MEME online tool. This analysis revealed 10 unique conserved motifs, labelled 1-10 (Figure 3). In Group 1, comprising 10 genes, we identified 10 conserved motifs. Most genes (6 out of 10) contained 10 motifs; however, genes showed different patterns. Genes *jg15324.t1* and *jg32849.t1* had nine motifs except motif 5; *jg22943.t1* had eight motifs except motifs 2 and 8; while *jg22944.t1* had only seven motifs, lacking motifs 4, 7, and 10. In contrast, genes *jg15324.t1* and *jg32849.t1* each possessed nine motifs, with motif five being the only one absent. In Group 2, comprising six genes, all identified 10 conserved motifs. The analysis of Group 3, which includes five genes, found that its members each exhibit 7 to 9 conserved motifs. Two of these genes, including *jg35863.t1* and *jg37538.t1* preserved the complete set of 9 motifs, while three genes, including *jg20243.t1*, *jg22092.t1*, and *jg38690.t1* displayed only seven motifs. In Group 4, comprising 11 genes, all identified nine conserved motifs. The analysis of Group 5, which contains nine genes, found that its members each exhibit 7 to 9 conserved motifs. Six of these genes preserved the complete set of nine motifs, while three genes displayed seven motifs. Remarkably, our findings indicate that 12 proteins conserved 10 motifs, while 21 proteins displayed nine motifs. Analyzing protein domains provided important insights into the biological roles of ARFs. Our analysis of the conserved domains in AmARF protein sequences revealed that every AmARF includes an auxin-responsive domain (Figure 3). To enhance our understanding of the structural diversity of *ARF* genes in *A. marina*, we examined their exon and intron arrangements. The exon/intron analysis showed that *AmARF* genes exhibited a range of 2 to 25 exons and 1 to 24 introns across various groups (Figure 3). Group 1 had 10–16 exons and 9–15 introns, group 2 had 14–25 exons and 13–24 introns, group 3 had 10–16 exons and 2–15 introns, group 4 had 13–17 exons and 12–16 introns, and group 5 had 2–6 exons and 1–5 introns. Furthermore, these genes have a remarkably conserved structure. Most genes in each of the five groups contained a surprising number of CDSs and introns, suggesting their activities might be very similar.

### 3.5. Examination of Cis-Regulatory Elements Within the Promoter Region of the AmARF Gene Family

**Figure 4:**
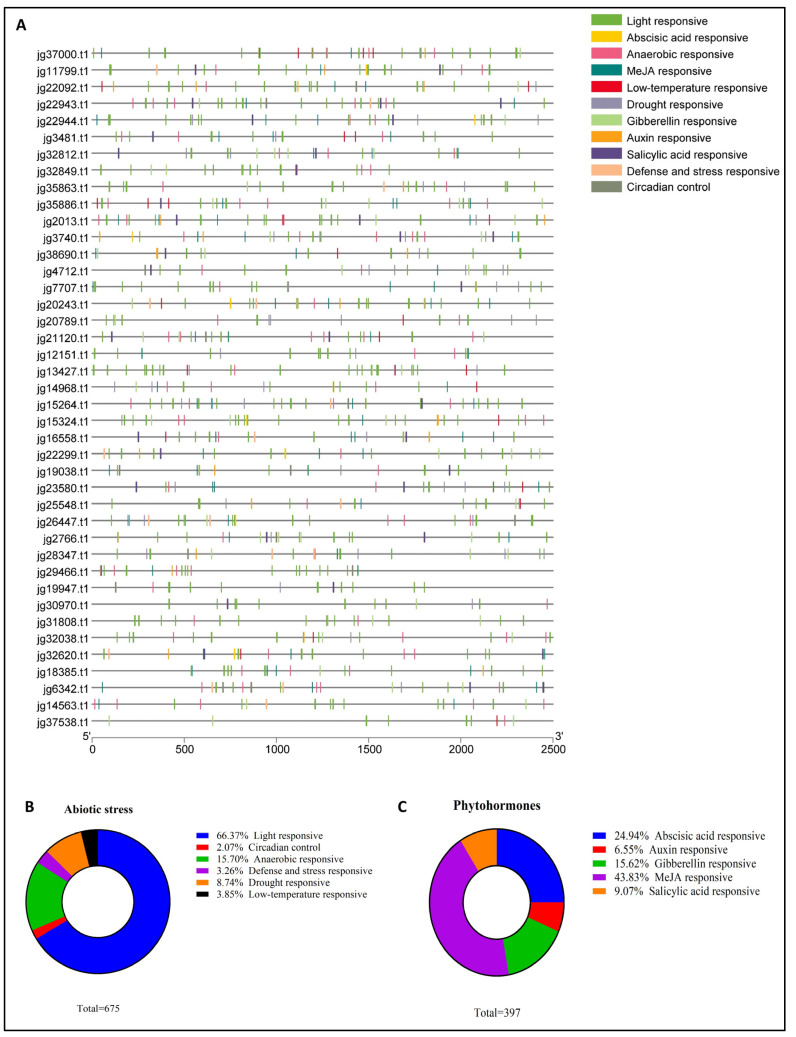
Exploration of cis-regulatory elements in *AmARF* promoter regions reveals various elements involved in responses to abiotic stress and phytohormones, indicated by different colors (**A**). The total number of *AmARF* genes containing elements related to abiotic stress (**B**) and phytohormone (**C**) responses is shown. Pie charts illustrate the percentage distribution of these elements within the abiotic stress-responsive and phytohormone-responsive groups. Unique colors represent specific elements and their proportions across *AmARF* genes.

The analyses of the cis-regulatory elements in the suspected promoter regions of *AmARF* genes aimed to enhance our understanding of their possible biological functions and regulatory networks. We identified 11 cis-acting elements in the 2500 bp upstream promoter region of the 41 *AmARF* genes. Additionally, we examined the distribution of hormone- and abiotic-stress-related cis-regulatory elements in the *AmARF* gene promoter region (Figure 4A). Cis-regulatory elements related to hormones (*n* = 397) include abscisic acid-responsive (24.9%): ABRE (*n* = 99); Methyl Jasmonate (MeJA)-responsive (43.83%): CGTCA-motif (*n* = 87), TGACG-motif (*n* = 87), and SARE (*n* = 1); Gibberellin-responsive (15.62%): GARE-motif (*n* = 30), P-box (*n* = 26), and TATC-box (*n* = 6); Salicylic acid-responsive (9.07%): TCA-element (*n* = 35); and Auxin-responsive (6.55%): AuxRE (*n* = 2), AuxRR-core (*n* = 7), TGA-box (*n* = 2), and TGA-element (*n* = 15). Auxin-responsive elements were found in the promoters of 19 *AmARF* genes. Both *jg15324.t1* and *jg38690.t1* had an AuxRE (TGTCTCAATAAG) in their promoters, whereas the promoters of *jg16558.t1*, *jg19038.t1*, *jg25548.t1*, *jg28347.t1*, *jg35863.t1*, *jg3740.t1*, and *jg38690.t1* featured an AuxRR-core (GGTCCAT). A distinct cis-element, the TGA-box (TGACGTAA), was discovered in the promoters of *jg23580.t1* and *jg37000.t1*. Additionally, the TGA-element (AACGAC) was detected in *jg22092.t1* (*n* = 4) and *jg2013.t1* (*n* = 2), as well as in *jg11799.t1*, *jg18385.t1*, *jg20243.t1*, *jg22299.t1*, *jg29466.t1*, *jg32038.t1*, *jg32620.t1*, and *jg3740.t1*. Cis-regulatory elements related to abiotic stress include light-responsive (66.37%): G-Box, TCT-motif, TCCC-motif, MRE, Sp1, LAMP-element, L-box, GTGGC-motif, I-box, GATT-motif, GT1-motif, Gap-box, GATA-motif, chs-CMA2a, GA-motif, Box 4, Box II, ATC-motif, ATCT-motif, AE-box, AT1-motif, 3-AF1 binding site, AAAC-motif, ACE, and chs-CMA1a; Circadian control (2.07%): circadian (*n* = 14); Anaerobic-responsive (15.70%): ARE (106); Defense and stress-responsive (3.26%): TC-rich repeats; Drought-responsive (8.74%): MBS (*n* = 59); and low-temperature response (3.84): LTR (*n* = 26) (Figure 4B,C). The results generally indicated that numerous elements related to abiotic stress and phytohormones were likely gene-specific and exhibited irregular expression patterns. As a result, genes containing these specific elements may serve as excellent candidates for further functional studies to uncover their protective functions during stress conditions and under phytohormone treatments.

### 3.6. Identification of miRNAs That Target AmARF Genes

**Figure 5:**
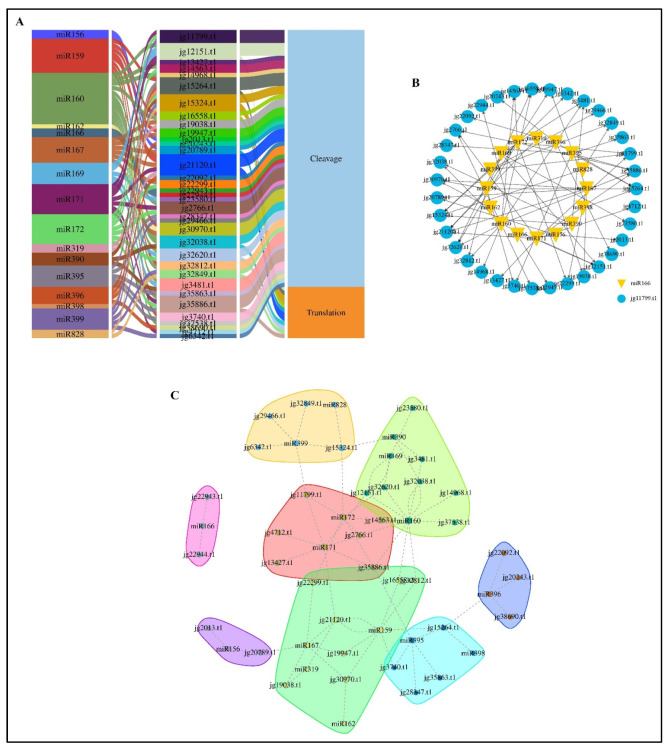
miRNA interactions with *AmARF* genes. (**A**) A Sankey diagram depicts the relationships between identified miRNAs, their target *AmARF* genes, and the predicted modes of inhibition, whether cleavage or translation. (**B**) The predicted miRNA–target interaction network illustrates the links between conserved miRNAs and *AmARF* genes. (**C**) Clustering analysis of miRNAs and their target genes based on correlation reveals regulatory modules within the *AmARF* miRNA interaction network. Different color boxes show different relationships among genes and miRNAs.

MicroRNAs (miRNAs) play crucial roles in managing plant growth, development, and stress responses. In this study, 73 miRNAs from 16 conserved families were identified as potential regulators of *AmARF* genes in *A. marina* (Figure 5). The Sankey diagram in Figure 5A illustrates their regulatory mechanisms: 61 miRNAs target 35 *AmARF* genes via cleavage, while 12 miRNAs control 10 *AmARF* genes through translation inhibition. Notably, 16 conserved miRNA families such as miR156, miR159, miR160, miR162, miR166, miR167, miR169, miR171, miR172, miR319, miR390, miR395, miR396, miR398, miR399, and miR828exert widespread regulatory influence over multiple *AmARF* genes.

Figure 5B illustrates a miRNA–target interaction network, emphasizing both conserved miRNAs and their associated *AmARF* targets. Meanwhile, Figure 5C groups miRNAs and target genes into functional clusters, exposing distinct regulatory modules within the *AmARF* network. These panels collectively demonstrate a highly interconnected regulation: for example, *jg21120.t1* is targeted by five miRNAs, and five other genes (*jg11799.t1*, *jg12151.t1*, *jg15264.t1*, *jg15324.t1*, and *jg35886.t1*) are each targeted by four miRNAs. The remaining *AmARF* genes are regulated by one to three miRNAs. These findings suggest that *AmARF* genes undergo extensive post-transcriptional regulation by both conserved and species-specific miRNAs. Confirming miRNA expression patterns and their target genes through experimental validation will be crucial to understanding their regulatory roles in *A. marina*.

### 3.7. Gene Ontology Enrichment Analysis

**Figure 6:**
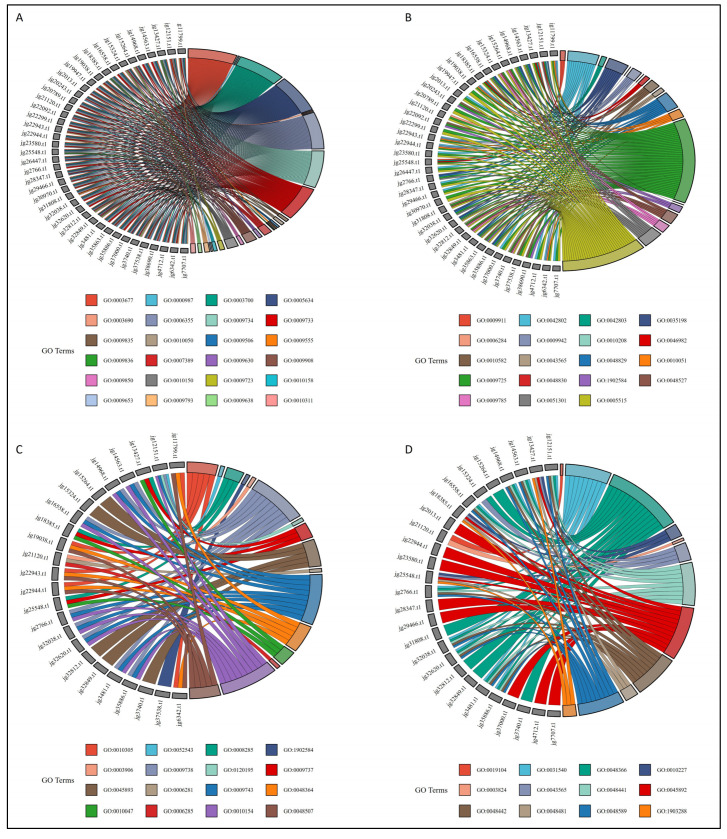
Chord analysis revealed 71 Gene Ontology (GO) terms linked to 41 *AmARF* genes, which are represented in (**A**) (GO terms 1–25), (**B**) (GO terms 26–44), (**C**) (GO terms 45–60), and (**D**) (GO terms 61–71). Refer to Supplementary Table S2 for detailed results.

**Figure 7:**
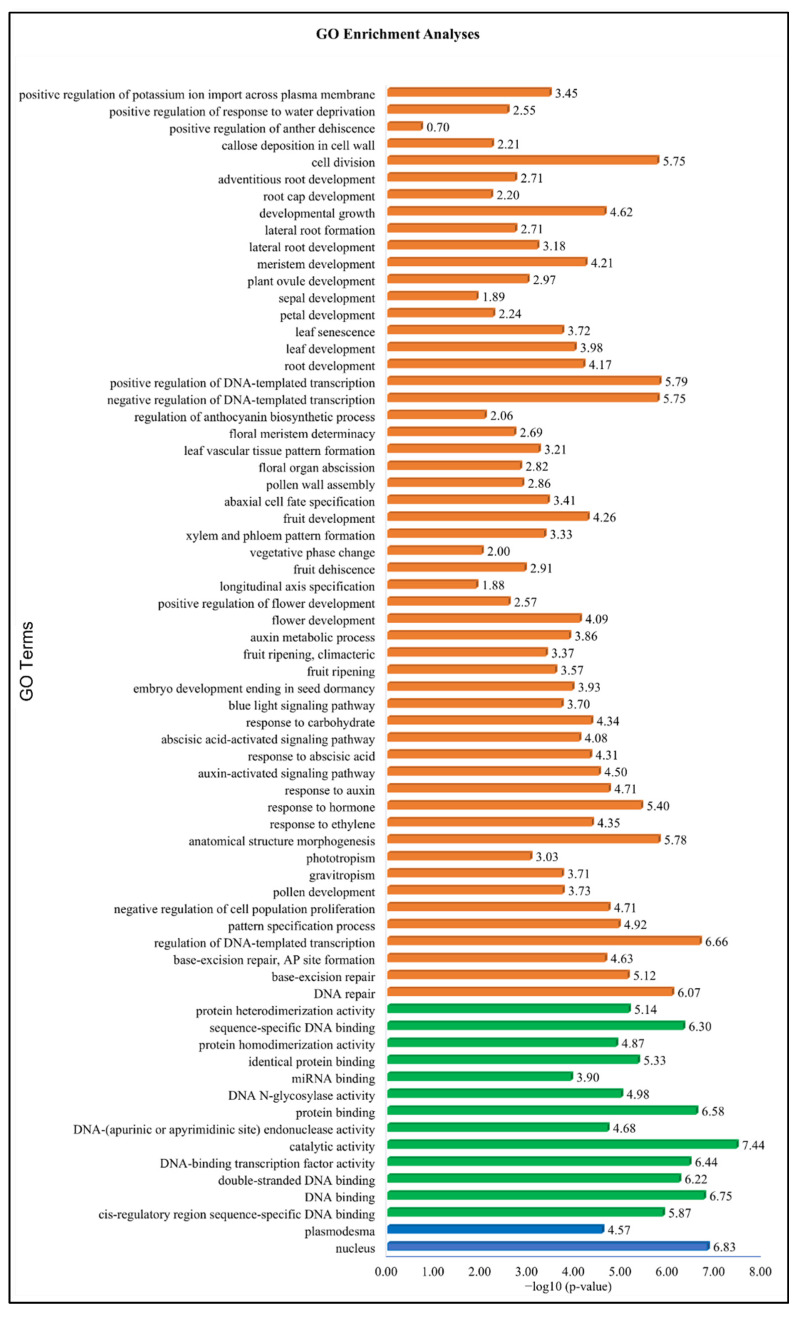
GO terms significantly enriched for *AmARF* genes, categorized into biological processes (orange), molecular functions (green), and cellular components (blue).

Before the GO enrichment analysis, we conducted a chord analysis and identified 71 Gene Ontology (GO) terms associated with 41 *ARF* genes. The chord plot in Figure 6 depicts the connections between these 71 GO terms and 41 genes, which are represented in Figure 6A (GO terms 1–25), Figure 6B (GO terms 26–44), Figure 6C (GO terms 45–60), and Figure 6D (GO terms 61–71). The chord analyses showed the six GO terms, including GO:0009725 (response to hormone), GO:0003677 (DNA binding), GO:0003700 (DNA-binding transcription factor activity), GO:0005634 (nucleus), GO:0006355 (regulation of DNA-templated transcription), and GO:0009734 (auxin-activated signaling pathway), were enriched in all 41 genes, while the other two GO terms including GO:0009733 (response to auxin) and GO:0005515 (protein binding) were enriched in 37 and 34 genes. To explore the functions of the AmARF family genes, we conducted Gene Ontology (GO) annotation and enrichment analyses of their biological processes (BP), molecular functions (MF), and cellular components (CC). The findings indicated that multiple GO terms were enriched in the BP, MF, and CC categories (Figure 7). The 41 AmARF proteins were associated with 69 GO terms, with a significant enrichment in BP (54) terms, followed by MF (13) and CC (02) (Supplementary Table S2). In the CC class, the significantly enriched terms included “nucleus” (GO:0005634) and “plasmodesma” (GO:0009506). In the MF enrichment, cis-regulatory region sequence-specific DNA binding (GO:0000987), DNA binding (GO:0003677), double-stranded DNA binding GO:0003690, DNA-binding transcription factor activity (GO:0003700), catalytic activity (GO:0003824), DNA-(apurinic or apyrimidinic site) endonuclease activity (GO:0003906), protein binding (GO:0005515), DNA N-glycosylase activity (GO:0019104), miRNA binding (GO:0035198), identical protein binding (GO:0042802), protein homodimerization activity (GO:0042803), sequence-specific DNA binding (GO:0043565), and protein heterodimerization activity (GO:0046982) were enriched. The number of abundantly enriched terms in the BP class was 54, as shown in Figure 7. In the BP enrichment, response to auxin (GO:0009733), auxin-activated signaling pathway (GO:0009734), cell division (GO:0051301), developmental growth (GO:0048589), leaf development (GO:0048366), leaf senescence (GO:0010150), response to hormone (GO:0009725), response to abscisic acid (GO:0009737), abscisic acid-activated signaling pathway (GO:0009738), blue light signaling pathway (GO:0009785), response to ethylene (GO:0009723), negative regulation of DNA-templated transcription (GO:0045892), positive regulation of DNA-templated transcription (GO:0045893), etc., were enriched (Supplementary Table S2). Overall, the results indicated that numerous elements associated with abiotic stress and phytohormones were likely gene-specific and exhibited irregular expression patterns. As a result, genes bearing these specific elements could serve as prime candidates for further functional experiments to uncover their protective roles under stress conditions and during phytohormone treatments.

### 3.8. ARFs Collinearity Analysis

**Figure 8:**
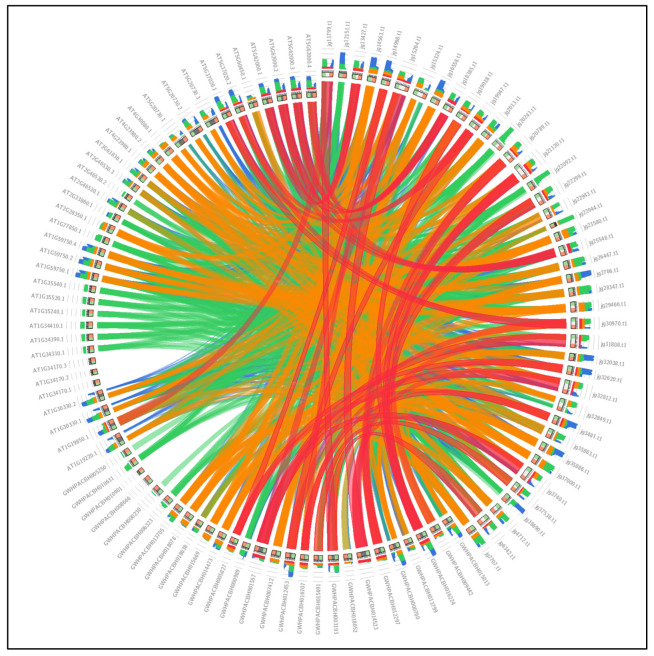
Collinearity analysis results among *Avicennia marina* (jg), *Kandelia obovata* (GWH), and *Arabidopsis thaliana* (AT). In the Circos plot, colors indicate similarity levels: blue for up to 25%, green for up to 50%, orange for up to 75%, and red for over 75%, reflecting their sequential identity in the explosion.

The comparative collinearity analysis included 41 ARF genes from *A. marina*, 27 from *K. obovata*, and 36 from *A. thaliana*. From the initial 3868 BLASTp alignments, applying a stringent E-value threshold (1 × 10^−10^) reduced the dataset to 984 high-confidence collinear gene pairs, which were used for visualization and interpretation. Between *A. marina* and *K. obovata*, we detected a greater number of collinear connections, as well as several blocks with relatively high bitscores (up to 1228.0). These blocks, represented by orange and red ribbons in the Circos plot, correspond to gene pairs with moderate to high sequence identity (>50%). This pattern suggests that many *ARF* genes in these two mangrove species retain detectable orthologous relationships, consistent with their closer evolutionary distance. In contrast, the synteny comparison between *A. marina* and *A. thaliana* revealed fewer homologous pairs, with most exhibiting low-to-moderate identity levels (≤50%), as indicated by blue and green ribbons. Only a small number of gene pairs showed higher similarity, and these largely corresponded to conserved core ARF domains rather than extended genomic segments. This result reflects the expected divergence between mangrove lineages and this distantly related terrestrial model plant. Overall, the combination of block counts, identity ranges, and bit scores indicates that mangrove species share more syntenic segments than with A. thaliana, aligning with their phylogenetic relationships. However, the patterns also show that large-scale genomic rearrangements have occurred across these lineages, with conserved segments mainly restricted to core gene regions (Figure 8).

### 3.9. Variation Across the ARF Family in Terms of 3D and Secondary Structure

**Figure 9:**
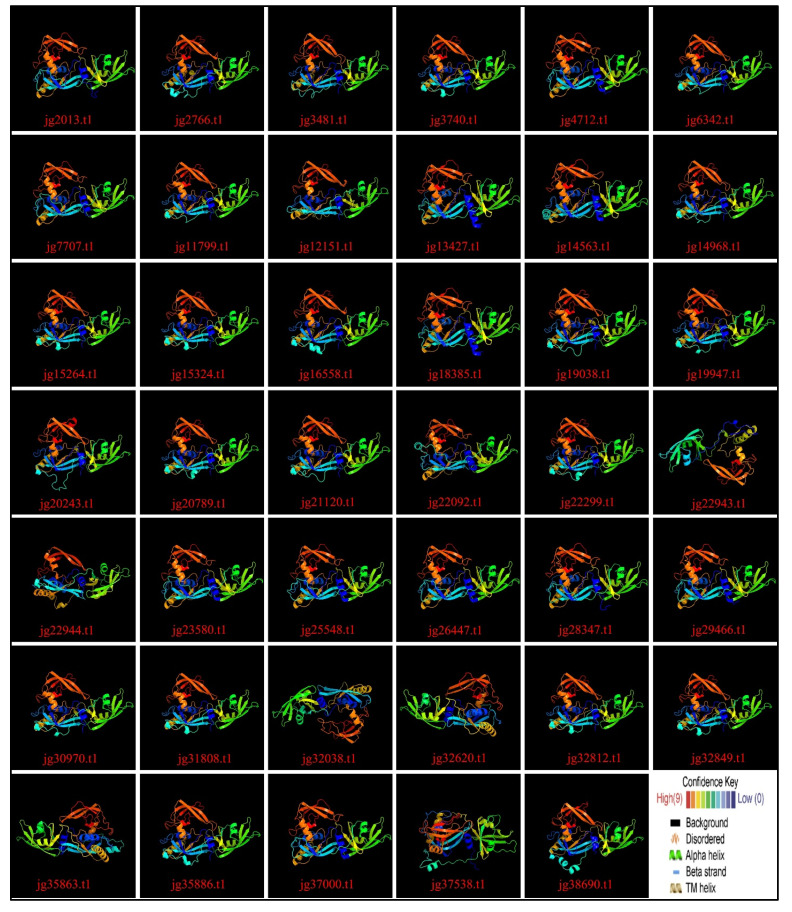
3D structures of proteins and their modeling. Refer to Supplementary Table S3 for detailed results.

Each AmARF protein was modeled in 3D (Figure 9), with detailed results of the chosen templates listed in Table S3. Four unique templates were identified, c4lduA, c4ldxB, c6sdgA, and c6ycqA, each with 100% confidence. The high confidence levels across multiple templates ensure reliable structural predictions. In total, 25 proteins were modeled using the “c4lduA” template, while 14 were modeled using the “c6ycqA” template. The remaining two proteins, jg35863.t1 and jg32038.t1, were modeled with the “c4ldxB” and “c6sdgA” templates, respectively. Their flexible, coil-rich structures may contribute to the functional versatility of AmARF proteins.

This secondary protein structure, typically including alpha helices, beta strands, disordered regions, and transmembrane (TM) helices, is detailed in Table S3. Among the 41 AmARF proteins, alpha helix content ranged from 2% (jg22092.t1) to 16% (jg21120.t1), beta-strand content from 9% (jg21120.t1) to 20% (jg7707.t1), TM helix percentage varied from 2% (jg23580.t1) to 6% (jg29466.t1), and disordered regions from 28% (jg22944.t1) to 69% (jg32812.t1). These results indicate a high structural adaptability in AmARF proteins, implying diverse functional roles. The wide range of secondary structure elements highlights their structural complexity and potential for adaptability.

### 3.10. Chromosomal Localization, Duplication Analysis, and Protein–Protein Interaction of the ARF Genes in A. marina

**Figure 10:**
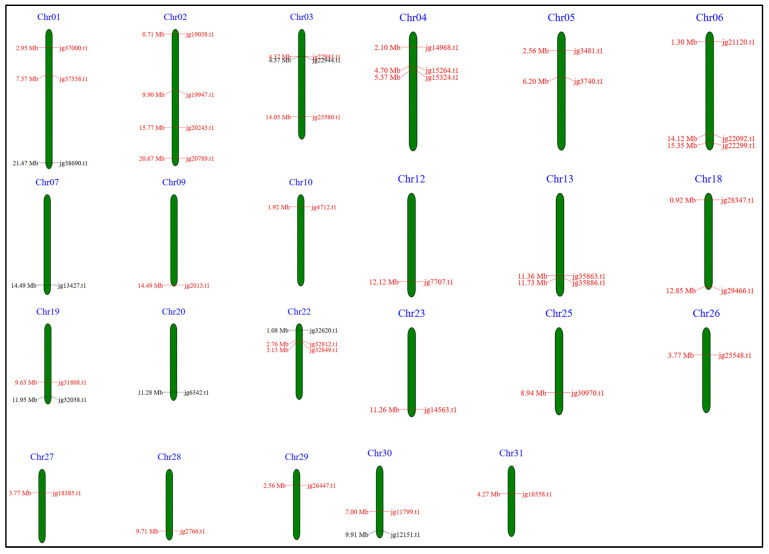
Chromosomal localization of *ARF* genes in *A. marina* is shown, with green numbers at the top of each chromosome indicating chromosome serial numbers. A total of 41 *AmARF* genes are unevenly distributed across 23 chromosomes and were mapped using the *A. marina* genome database with MapGene2Chromosome (MG2C). Chromosome lengths are scaled in megabases (Mb). The positions of *ARF* genes are listed on the left, while their gene names are on the right.

**Figure 11:**
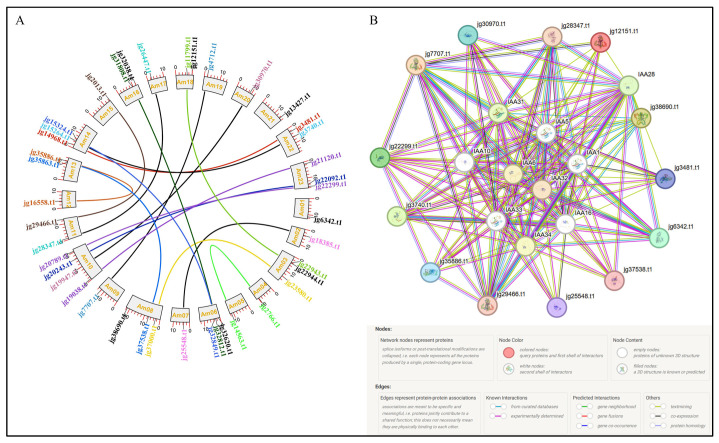
Duplication and interaction analysis of *AmARF* genes. (**A**) Circos diagram illustrating *ARF* gene duplication within the *A. marina* genome. Different lines and colors denote segmental and tandem duplications among connected *AmARF* genes. (**B**) Schematic diagram displaying the protein–protein interaction network of ARF proteins, generated using the STRING database.

The latest assembly of the *A. marina* genome presents a chromosome-level structure comprising 32 super scaffolds, each corresponding to a chromosome. The *ARF* genes in *A. marina* are located solely on chromosome 23 (Figure 10). Chromosome 02 of *A. marina* had the most *ARF* genes, with 4, whereas chromosomes 01, 03, 04, 06, and 22 each had 3. In comparison, chromosomes 5, 13, 18, 19, and 30 each carried two *ARF* genes, whereas the other chromosomes—7, 9, 10, 12, 20, 23, 25, 26, 27, 28, 29, and 31—carried only one. Most chromosomes harboured 1–3 *ARF* genes, suggesting a relatively uniform distribution of *AmARF* genes across all chromosomes. To identify the evolution of the *AmARF* gene expansion, gene duplication events were analyzed using MEGA12. A total of 17 gene pairs were identified as duplicated within the AmARF gene family (Figure 11A). Importantly, all 17 gene pairs were part of segmental duplications, with no evidence of tandem duplication. We calculated the Ka/Ks values for 17 duplicated *AmARF* gene pairs to analyze selection. The findings revealed that the Ka/Ks ratios ranged from 0.194 to 0.301. In 17 pairs of *AmARF* members, the Ka/Ks ratio was below 1, indicating that all *AmARF* duplicated genes experienced purifying selection (adverse selection) (Table 2). All 41 AmARF members interacted with identified proteins and were grouped, likely indicating distinct functions. Our interactome analysis validated their findings, showing that ARF interacts with IAA proteins (including IAA1, IAA5, IAA6, IAA10, IAA16, IAA31, IAA32, IAA33, and IAA34), and their activities may influence one another. Different lines of color among all proteins correspond to various interactions. In short, these findings suggest that AmARF proteins interact strongly with diverse IAA members, potentially allowing them to perform complex biological functions (Figure 11B).

### 3.11. Expression Analysis of ARF Genes in A. marina Leaves Under Salt and Auxin Stresses.

**Figure 12:**
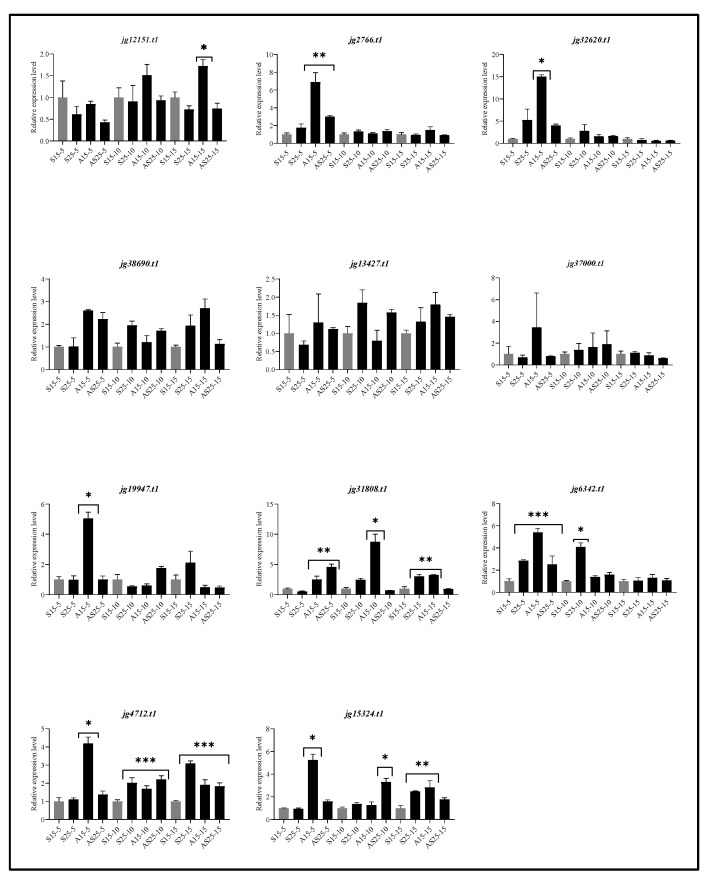
Expression profiles of 11 representative *AmARF* genes in seedling-stage *A. marina* leaves under salt and IAA treatments. Relative transcript levels were measured by qRT-PCR after 5, 10, and 15 days under four conditions: 15‰ salt (S15, control), 25‰ salt (S25), 50 µM IAA with 15‰ salt (A15), and 50 µM IAA with 25‰ salt (AS25). The 11 ARFs were selected to represent all major phylogenetic clades and to include genes with distinct regulatory features. Data are presented as mean ± SD of three biological replicates; significant differences are shown (*p* < 0.05, *p* < 0.01, *p* < 0.001). * = Displayed significant differences, * *p* < 0.05, ** means *p* < 0.01, *** means *p* < 0.001. Light grey colour shows control, and black colour shows different treatments.

We selected 11 representative *AmARF* genes (*jg12151.t1*, *jg2766.t1*, *jg32620.t1*, *jg38690.t1*, *jg13427.t1*, *jg37000.t1*, *jg19947.t1*, *jg31808.t1*, *jg6342.t1*, *jg4712.t1*, and *jg15324.t1*) for expression analysis to capture the functional and evolutionary diversity of the ARF family rather than analyzing all 41 genes. These 11 genes were chosen because they span all major phylogenetic clades, differ in domain composition, and include *ARFs* predicted to interact with multiple conserved miRNAs, making them strong candidates for mediating hormonal and salt-stress responses. Their expression patterns revealed clear treatment-specific regulatory trends. Under moderate salt stress (S25), several genes (e.g., *jg2766.t1*, *jg32620.t1*, *jg19947.t1*) showed strong early upregulation at 5 days, indicating rapid activation of auxin-related signaling, while others, such as *jg38690.t1* and *jg37000.t1* remained relatively stable (Figure 12). In contrast, exogenous IAA under non-stress conditions (A15) induced higher expression in most genes, especially jg31808.t1 and jg4712.t1 reflecting their sensitivity to auxin supply. When IAA was combined with salt stress (AS25), several genes (*jg12151.t1*, *jg32620.t1*, *jg31808.t1*, *jg6342.t1*, *jg15324.t1*) exhibited stronger induction than in either treatment alone, suggesting a synergistic role of auxin in enhancing the salt-response pathway. Across the time course, early responses (day 5) were typically more pronounced than those at days 10 and 15, indicating initial transcriptional activation followed by stabilization or feedback regulation. Overall, the diverse expression patterns show that these selected *AmARF* genes function as both positive and negative regulators during salt and auxin responses, supporting their relevance for understanding the hormonal regulation mechanisms in *A. marina*.

## 4. Discussion

Auxin plays a crucial role in plant growth and development. *ARFs* are vital components of the auxin signaling pathway; thus, they can regulate the transcription of auxin-responsive genes involved in plant growth stages, development, and stress responses [35]. Plant homeostatic and stress-response mechanisms are well-established and can account for the unanticipated variations in endogenous IAA levels between treatments. Even though the A15 treatment included exogenous IAA, plants strictly control auxin homeostasis, and external application frequently sets off negative feedback mechanisms that lower rather than raise free IAA concentration. These include increased conjugation of IAA to amino acid conjugates (IAA-Asp, IAA-Glu), inhibition of endogenous auxin production, and overexpression of IAA-oxidase/DAO enzymes [12,36]. In addition, IAA is chemically unstable and can be rapidly metabolized or inactivated before it accumulates in leaf tissues [37]. Auxin dynamics become significantly more complicated under salt stress: salinity modifies intracellular auxin gradients, speeds up IAA breakdown, and affects polar auxin transport by influencing PIN and AUX/LAX transporters [38]. Even under IAA irrigation, these mechanisms contribute to the explanation of why endogenous IAA decreased at specific times. Furthermore, the significant rise in ABA under moderate salt (S25) is in line with its essential function in stress signaling and osmotic adjustment [39]. ABA is recognized for its capacity to inhibit auxin transit and stability, hence further diminishing free IAA levels in stressed leaves [40]. Together, these mechanisms indicate that *A. marina* does not simply accumulate auxin when supplied exogenously; instead, it actively modulates auxin metabolism and transport to maintain hormonal balance under salt stress. Thus, the observed hormone patterns reflect a coordinated auxin–ABA regulatory response rather than a direct linear effect of IAA application. Thus, to elucidate the role of *A. marina* ARFs in specific auxin responses, we used the *A. marina* genome to conduct a comprehensive genome-wide survey of the ARF gene family. In this study, 41 *A. marina ARF* genes were identified and named based on their chromosomal positions. In this study, 41 *A. marina* ARF genes were identified through genome-wide analysis. The total number of ARF genes in *A. marina* is comparable to that of kiwifruit, which also contains 41 *ARFs* [17], and is higher than those reported in other woody species such as *Populus trichocarpa* (35) [7], 29 in Mango (*Mangifera indica* L.) [18], and 17 in Peach (*Prunus persica* L.) [20]. This relatively larger number of *ARFs* in *A. marina* may be attributed to species-specific gene duplication events and the unique genomic evolution of mangroves. When compared with herbaceous models, the number of *AmARFs* also exceeds the *ARF* counts documented in tomato (17 *SlARFs*) [24] and rice (25 *SbARFs*) [41].

The phylogenetic analysis revealed that *ARF* genes from *Avicennia marina* (jg), *Kandelia obovata* (GWH), and *Arabidopsis thaliana* (AT) clustered into four major groups. This analysis also indicated that *AmARF* and *Kandelia obovata* are the most closely related. Our results align with previous research, as seen in the cases of alfalfa (Medicago sativa) [16], Pineapple (*Ananas comosus*) [42], and *Brachypodium distachyon* L. [43]. Promoter sequences with cis-regulatory motifs help us understand how AmARF genes respond to environmental factors. Among them were those that responded to phytohormones and stress response cis-elements. Our phylogenetic analysis suggests that the four groups represent ancient gene duplications that led to functional diversification within the ARF family. Earlier research indicates that ARFs are grouped into conserved evolutionary clades, each with unique regulatory roles, such as activators, repressors, and specialized regulators [10,44]. Our Group 1 mainly includes classical transcriptional activators characterised by well-conserved MR and CTDs [45]. Group 2 consists of both activators and repressors and represents the largest and most functionally diverse clade, in line with earlier comparative studies reporting lineage-specific expansions in this group [41]. Group 3 includes more variable repressors with altered MRs, which have been associated with context-specific auxin responses [46]. Group 4 comprises ARFs with altered or shortened CTDs, which might operate via non-traditional auxin signalling pathways [47]. Overall, the grouping pattern is consistent across all three species examined and aligns with the well-known ARF evolutionary structure, suggesting that these clades are conserved functional lineages rather than artefacts of tree construction.

Furthermore, 41 *AmARF* genes whose promoters contain a cis-element related to responding to IAA and stress. Our result is consistent with a previous finding: promoter cis-elements reportedly have a significant influence on the transcriptional control of genes in plants during stress and IAA treatments [7]. An analysis of 41 genes across three groups revealed significant genomic diversity, characterized by variations in motif composition, exon–intron structure, and functional domains. However, the number of motifs varied among genes, specific conserved motifs, such as Motif 2 of gene *jg11799.t1* appeared twice in Group 1, while Motif 3 of gene *jg32038.t1* was identified twice in Group 3, indicating their potential regulatory significance. Moreover, the discrepancies in exon–intron organization suggested possible evolutionary divergence or mechanisms of alternative splicing. Importantly, domain analysis revealed auxin response domains in all 41 genes, linking these to auxin signaling pathways. These findings collectively showcase conserved and unique genomic features that may contribute to functional specialization in biological processes, especially in auxin-mediated responses. Future research could examine how variations in motifs and gene structures affect gene expression and protein functions within these pathways [17].

There is increasing evidence that microRNAs regulate ARF transcript abundance through posttranscriptional mechanisms [48]. Recently, microRNAs (miRNAs) have been found to play a role in how plants respond to environmental stress by regulating genes involved in auxin signaling [28,29]. In Arabidopsis, miR167 targets *AtARF6/8*, while miR160 targets *AtARF10/16/17* [30,31]. miR160 and miR167 play crucial roles in supporting normal development under stressful conditions by regulating the expression of targeted *ARF* genes [32]. Our analysis identified that *jg19038.t1*, *jg19947.t1*, *jg20789.t1*, *jg21120.t1*, *jg22299.t1*, and *jg30970.t1* likely have target sites for miR167, while 11 genes have potential sites for miR160. MicroRNA160 and microRNA167 target sites were found in certain maize *ZmARF* genes [49]. Similarly, in tomatoes, *Sl-ARF6A*, *Sl-ARF8A*, and *Sl-ARF8B* are predicted targets of miR167 [50]. The transcript levels of tomato *Sl-ARF10A*, *Sl-ARF10B*, *Sl-ARF16A*, *Sl-ARF16B*, and *Sl-ARF17* are interestingly regulated by miR160, highlighting a fascinating area of research in plant genetics [50]. These studies demonstrate that *ARF* genes play a crucial role in enabling plants to grow and develop, as well as in their response to environmental stresses.

Multiple studies have confirmed that *AmARFs* are involved in various physiological responses to salt and IAA stress. This research aimed to investigate the impact of salt and IAA stress on the expression of the *AmARF* gene. Results showed that *AmARF* gene expression increased and decreased under salt and IAA stress compared to the control. Since *ARF* genes are essential in regulating phytohormone signalling, understanding the function of each family member is important. ARF transcription factors are vital for plant growth and development and play an active role in plant stress resistance [16,51,52].

Additionally, *AcARF5* helps improve the ability of kiwifruit (*Actinidia chinensis*) to withstand salt and drought stress, making the plant more resilient [17]. The concentration of auxin regulates the activity of ARFs [15,43], so we focused on the effect of IAA hormones on *ARF* expression. Almost all *AmARFs* were significantly upregulated in *A. marina* leaves following IAA treatment. Interestingly, *SbARF* genes were strongly induced by salt stress in sorghum leaf tissues, corroborating the evidence that most *StARF* genes were induced upon abiotic stresses [53,54]. These findings offer a valuable foundation for identifying candidate genes that regulate plant growth and architecture under stress, enabling future functional validation through CRISPR/Cas-mediated knockout, overexpression, or other genome-editing strategies, which may ultimately support genetic improvement efforts in *A. marina* and other crops.

## 5. Limitations and Future Directions

While this research offers an initial genome-wide overview of the ARF gene family in *A. marina*, several limitations must be considered. First, many findings are based on computational predictions, such as promoter cis-elements, miRNA targeting, and protein interactions, which need further experimental validation. Second, physiological and expression studies were limited to leaf tissues and might not reflect responses in roots, stems, or reproductive organs. Third, only a selection of *AmARFs* was confirmed through qRT-PCR, and hormone levels were assessed with limited HPLC validation. Lastly, uncertainties related to the current *A. marina* genome assembly could impact the precision of gene models and annotations.

To address these challenges, future research should include broad transcriptomic profiling (RNA-Seq) under various stress conditions and employ promoter–reporter assays (GUS/LUC) to identify regulatory elements. Validating transcriptional and protein interactions through Y1H, Y2H, and Co-IP will help elucidate molecular networks. Moreover, 5′ RACE should be used to confirm miRNA–AmARF interactions, and functional experiments with CRISPR/Cas-mediated knockout or overexpression are necessary to clarify the roles of key *AmARFs* in hormonal regulation and salt–auxin signaling. These strategies will advance the current foundational work toward a detailed mechanistic understanding of ARF-driven stress adaptation in *A marina*.

## 6. Conclusions

This is the first study to perform genome-wide identification and analysis of ARF family members in *Avicennia marina*. In this study, 41 *AmARF* genes were identified and distributed unevenly across 23 chromosomes. The identified AmARFs are categorized into four groups, each with highly similar gene structures and motif patterns. Cis-element prediction suggests that *AmARFs* are probably triggered by environmental cues, hormones, and cellular development processes. Multiple analyses were conducted to explore the evolution of the ARF gene family in the *A. marina* genome. These included gene identification, subcellular localization, phylogenetic analysis, chromosomal mapping, microRNA targeting, gene ontology, domain and 3D structural variations, synteny and duplication assessments, gene structure and motif analysis, cis-regulatory element investigation, and expression profiling under varied salt and IAA conditions. Based on the expression profiles, 11 *AmARF* genes are specifically expressed in leaves and primarily contribute to salt and IAA resistance under stress. These findings will facilitate the identification of potential genes that enhance plant architecture in response to stressors, supporting future breeding and genetic improvements of *AmARF* genes in various crops. Such approaches may include CRISPR/Cas-mediated deletion, overexpression, and other genetic technique changes.

## Figures and Tables

**Table 1:** Overview of 41 *AmARF* genes and their essential physicochemical properties.

Sequence ID	AA ^1^	CDS ^2^	MW ^3^	pI ^4^	II ^5^	AI ^6^	GRAVY ^7^	SL ^8^
*jg11799.t1*	923	2769	102,030.6	5.3	56.34	73.92	−0.432	nucleus
*jg12151.t1*	621	1863	67,860.82	6.74	40.79	82.29	−0.241	nucleus
*jg13427.t1*	801	2403	89,332.21	6.18	61.23	64.87	−0.663	nucleus
*jg14563.t1*	691	2073	76,248.19	6.56	44.43	74.79	−0.385	nucleus
*jg14968.t1*	693	2079	75,977.67	7.21	49.44	69.06	−0.41	nucleus
*jg15264.t1*	1142	3426	124,981.77	6.16	63.77	75.9	−0.505	nucleus
*jg15324.t1*	1086	3258	120,456.08	6.32	61.65	75.81	−0.566	nucleus
*jg16558.t1*	700	2100	77,331.65	6.39	51.84	75.91	−0.362	nucleus
*jg18385.t1*	782	2346	87,712.9	6.52	62.6	68.57	−0.623	nucleus
*jg19038.t1*	819	2457	91,062.47	6.32	58.67	72.53	−0.451	nucleus
*jg19947.t1*	808	2424	90,706.42	6.26	58.04	72.86	−0.422	nucleus
*jg2013.t1*	713	2139	79,216.69	6.51	53.32	74.1	−0.426	nucleus
*jg20243.t1*	854	2562	93,784.01	8.61	48.58	71.57	−0.355	nucleus
*jg20789.t1*	868	2604	95,306.8	6.46	61.51	78.73	−0.301	nucleus
*jg21120.t1*	1264	3792	139,295.27	7	57.41	77.23	−0.391	nucleus
*jg22092.t1*	676	2028	74,134.34	8.24	55.2	68.93	−0.412	nucleus
*jg22299.t1*	954	2862	104,921.35	6.72	63.82	75.41	−0.401	nucleus
*jg22943.t1*	810	2430	89,317.38	5.53	48.96	77.38	−0.393	nucleus
*jg22944.t1*	361	1083	40,412.34	9.39	54	77.84	−0.356	nucleus
*jg23580.t1*	685	2055	76,736.46	7.36	55.45	70.85	−0.558	nucleus
*jg25548.t1*	803	2409	89,606.21	6.23	58.22	65.69	−0.584	nucleus
*jg26447.t1*	663	1989	75,569.74	6.59	59.67	75.84	−0.555	nucleus
*jg2766.t1*	631	1893	69,408.41	6.26	43.55	75.69	−0.369	nucleus
*jg28347.t1*	680	2040	76,174.1	5.76	58.08	74.41	−0.429	nucleus
*jg29466.t1*	833	2499	93,139.23	6.7	52.73	79.06	−0.328	nucleus
*jg30970.t1*	910	2730	102,132.19	6.39	59.29	70.92	−0.469	nucleus
*jg31808.t1*	1068	3204	117,372.04	6.15	66.7	72.41	−0.544	nucleus
*jg32038.t1*	586	1758	64,740.11	8.92	50.45	68.05	−0.452	nucleus
*jg32620.t1*	678	2034	73,752.22	6.71	46.51	71.19	−0.346	nucleus
*jg32812.t1*	1105	3315	121,212.97	6.15	63.25	73.87	−0.498	nucleus
*jg32849.t1*	1016	3048	112,319.7	6.36	66.61	70.94	−0.598	nucleus
*jg3481.t1*	691	2073	75,413.34	7.25	49.72	73.95	−0.313	nucleus
*jg35863.t1*	769	2307	85,467.36	6.31	56.8	73.25	−0.482	nucleus
*jg35886.t1*	700	2100	76,452.86	7.9	42.97	75.33	−0.314	nucleus
*jg37000.t1*	744	2232	83,240.88	6.11	58.31	72.57	−0.491	nucleus
*jg3740.t1*	1097	3291	120,835.52	6.12	60.44	75.63	−0.497	nucleus
*jg37538.t1*	783	2349	86,909.13	5.72	53.41	71.7	−0.466	nucleus
*jg38690.t1*	639	1917	70,890.23	6.89	60.48	75.23	−0.395	nucleus
*jg4712.t1*	825	2475	92,646.74	8.67	56.23	75.02	−0.451	nucleus
*jg6342.t1*	935	2805	103,811.84	5.94	47.51	74.35	−0.411	nucleus
*jg7707.t1*	675	2025	75,633.85	7.1	60.03	73.47	−0.482	nucleus

AA ^1^: Number of amino acids; CDS ^2^: Coding Sequence; MW ^3^: Molecular weight; pI ^4^: Isoelectric point; II ^5^: Instability Index; AI ^6^: Aliphatic Index; GRAVY ^7^: Grand average of hydropathicity; SL ^8^: Subcellular Localization.

**Table 2:** Ka/Ks analysis of *AmARF* genes.

Gene 1	Gene 2	Ka	Ks	Ka/Ks
*jg11799.t1*	*jg22943.t1*	0.110	0.510	0.215
*jg15324.t1*	*jg32849.t1*	0.163	0.615	0.265
*jg15264.t1*	*jg3740.t1*	0.111	0.539	0.207
*jg31808.t1*	*jg32812.t1*	0.125	0.543	0.230
*jg19947.t1*	*jg30970.t1*	0.096	0.417	0.230
*jg19038.t1*	*jg21120.t1*	0.081	0.417	0.194
*jg20789.t1*	*jg22299.t1*	0.111	0.455	0.243
*jg20243.t1*	*jg22092.t1*	0.160	0.592	0.271
*jg35863.t1*	*jg37538.t1*	0.146	0.581	0.252
*jg18385.t1*	*jg25548.t1*	0.138	0.658	0.210
*jg26447.t1*	*jg28347.t1*	0.179	0.594	0.301
*jg2013.t1*	*jg29466.t1*	0.131	0.457	0.286
*jg23580.t1*	*jg37000.t1*	0.141	0.512	0.275
*jg4712.t1*	*jg7707.t1*	0.144	0.530	0.272
*jg16558.t1*	*jg35886.t1*	0.152	0.722	0.210
*jg14563.t1*	*jg2766.t1*	0.115	0.589	0.195
*jg14968.t1*	*jg3481.t1*	0.128	0.530	0.242

## Data Availability

Data pertaining to the study have been included in the article or as Supplementary Material; further inquiries can be directed to the corresponding author.

## Supplementary Materials

The following supporting information can be downloaded at: https://www.mdpi.com/article/10.3390/biology14121774/s1, Figure S1. Analysis of the transmembrane domain across all AmARF members. The horizontal axis indicates the amino acid sequence, while the vertical axis shows the probability that the region is part of the protein. The purple line denotes the transmembrane region, the blue line indicates the inside of the membrane, and the orange line marks the outside of the membrane; Table S1. Protein pairwise similarity matrix; Table S2. GO enrichment analysis; Table S3. Information on predicted protein 3D structures and modeling; Table S4. List of *AmARF* genes based on Pfam (PF06507); Table S5. Three species protein sequences for the phylogenetic tree; Table S6. Details of the primer sequences.
