# Supplementary figures and images for: Genome-Wide Discovery and Characterization of the Auxin Response Factor (ARF) Gene Family in Avicennia marina That Regulates Phytohormone Levels and Responds to Salt and Auxin Treatments
Source: Biology (Basel). 2025 Dec 11;14(12):1774. doi: 10.3390/biology14121774 (PMC12730682; doi:10.3390/biology14121774)

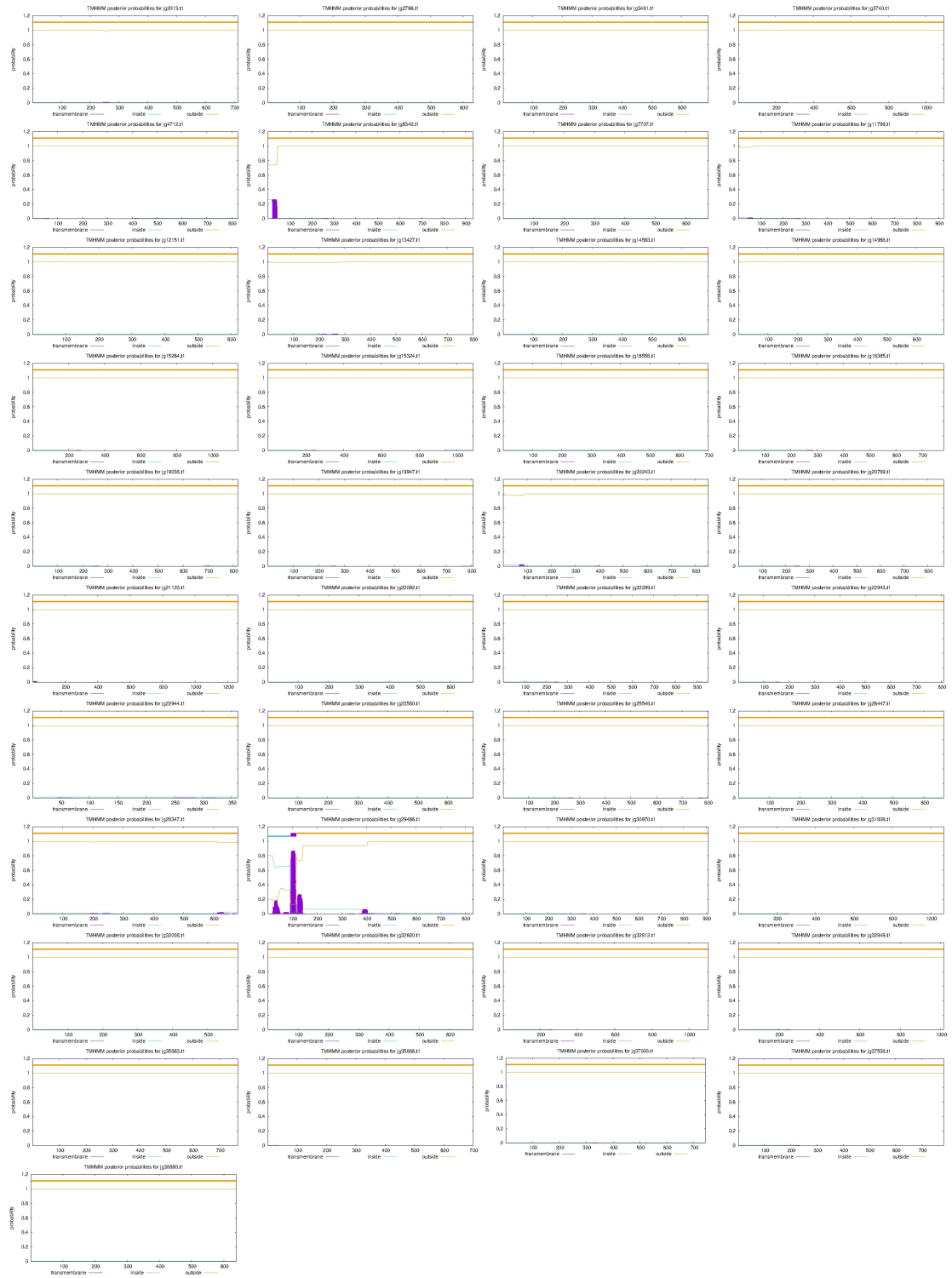

Supplement: Supplementary file 1 [file biology-14-01774-s001.zip › Supplementary Figure and Tables/Supplementary Figure.pdf]
